# Risks at the DNA Replication Fork: Effects upon Carcinogenesis and Tumor Heterogeneity

**DOI:** 10.3390/genes8010046

**Published:** 2017-01-22

**Authors:** Tony M. Mertz, Victoria Harcy, Steven A. Roberts

**Affiliations:** School of Molecular Biosciences, College of Veterinary Medicine, Washington State University, Pullman, WA 99164, USA; mertztony@vetmed.wsu.edu (T.M.M.); vharcy@vetmed.wsu.edu (V.H.)

**Keywords:** replication, mutagenesis, cancer, APOBEC, mismatch repair, polymerase delta, polymerase epsilon, replication stress, nucleotide pools

## Abstract

The ability of all organisms to copy their genetic information via DNA replication is a prerequisite for cell division and a biological imperative of life. In multicellular organisms, however, mutations arising from DNA replication errors in the germline and somatic cells are the basis of genetic diseases and cancer, respectively. Within human tumors, replication errors additionally contribute to mutator phenotypes and tumor heterogeneity, which are major confounding factors for cancer therapeutics. Successful DNA replication involves the coordination of many large-scale, complex cellular processes. In this review, we focus on the roles that defects in enzymes that normally act at the replication fork and dysregulation of enzymes that inappropriately damage single-stranded DNA at the fork play in causing mutations that contribute to carcinogenesis. We focus on tumor data and experimental evidence that error-prone variants of replicative polymerases promote carcinogenesis and on research indicating that the primary target mutated by APOBEC (apolipoprotein B mRNA-editing enzyme catalytic polypeptide-like) cytidine deaminases is ssDNA present at the replication fork. Furthermore, we discuss evidence from model systems that indicate replication stress and other cancer-associated metabolic changes may modulate mutagenic enzymatic activities at the replication fork.

## 1. Introduction

The important task of copying genetic information during each cell division is accomplished through DNA replication. Normal DNA replication is phenomenally accurate. Estimates of the mutation rate per base pair during each replication cycle range from 10^−9^ (based on exome sequencing of somatic cells and estimation of cell division based on telomere length) [1] to 10^−10^ (based on mutations accumulated in individual loci) [2]. The fidelity of DNA replication is contingent upon the very high base selectivity of replicative polymerases delta (Polδ) and epsilon (Polε) during dNTP incorporation, the ability of these polymerases to proofread errors using their exonuclease domains, and error-correction by mismatch repair (MMR). In addition, maintenance of proper dNTP pools and an undamaged template are instrumental in minimizing polymerase errors during replication.

Genetic and epigenetic changes within cells that increase the number of errors that occur during DNA replication have many consequences. Mutations introduced during DNA replication provide the genetic basis for phenotypic variation upon which natural selection acts during the process of evolution. However, most mutations that affect protein function are deleterious in nature. Therefore, mutations that reduce replication fidelity in unicellular organisms and in germline cells of multicellular organisms tend to reduce fitness. Extremely inaccurate DNA replication can lead to a rapid accumulation of mutations that disrupts cellular processes needed for viability and extinguish clonal populations of cells within several generations [3,4].

Mutations or dysregulated enzymatic activities that decrease replication fidelity to non-lethal levels increase the likelihood by which loss- and gain-of-function mutations occur and thereby have the potential to indirectly alter many cellular processes. In somatic cells, the establishment of an elevated mutation rate (termed a mutator phenotype) has been proposed to be a key step in the progression of many cancers [5]. This hypothesis is supported by observations that genomic instability is both a common and defining characteristic of cancer. Cells with elevated levels of genomic instability have an increased likelihood to acquire genetic changes that result in the loss of tumor suppressors and/or activation of oncogenes. Both chromosomal instability (loss and gain of entire chromosomes, translocations, and large deletions and duplications) and point mutation instability (deletions, insertions, and base substitutions that typically involve one to three base pairs) contribute to key driver mutations leading to carcinogenic transformation. While it is becoming increasingly clear that cancer cells of many tumor types have elevated rates of mutation [5,6], the molecular basis for the mutator phenotype in many tumors is not fully understood. Here, we review literature indicating that a subset of tumors contains an elevated number of base pair substitutions caused by loss of proofreading capacity and DNA repair activities as well as increased DNA damage at the replication fork.

### 1.1. An Overview of the Eukaryotic DNA Replication Fork

**Figure 1:**
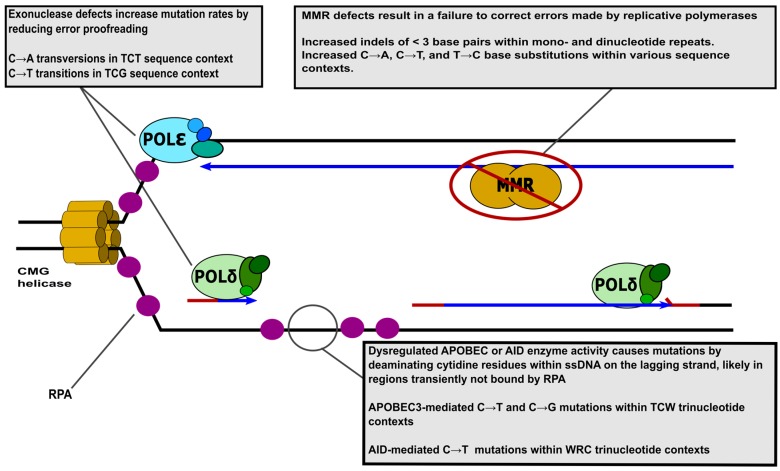
Replication fork structure and mutagenic changes in enzyme activity. Replicative DNA polymerases Polδ (green) and Polε (blue) are shown on the lagging and leading strands, respectively. ssDNA binding protein RPA is depicted as purple circles. The template DNA stands, RNA primers, and newly synthesized daughter stands are represented by black, red, and blue lines, respectively. Please note that simplified depictions of proteins do not convey structural information and are not to scale. The grey call-out boxes describe mutagenic activities at the replication fork and associated mutation signatures from human tumors. Several important proteins present at the replication fork, the Replication factor C (RFC) complex, proliferating cell nuclear antigen (PCNA), and Polα have been omitted for the sake of simplicity. W (either A or T), R (either A or G).

The basic unit of DNA replication is the replication fork, at which DNA is denatured and copied. Two replication forks commence DNA replication at most origins of replication. In *Saccharomyces cerevisiae*, replication origins are defined by specific autonomous replicating sequences (ARS) [7,8]. The total number of *S. cerevisiae* replication origins is in the range of 300 to 400 with a slightly smaller number being utilized for each genome replication event [9]. Larger mammalian genomes employ approximately 40,000 origins [10]. The elements that represent human origins of replication and pathways that determine usage and timing are still poorly understood (reviewed in [11,12,13]). DNA replication is initiated by the action of the origin recognition complex (ORC), which binds to replication origins and serves as the cornerstone from which the pre-replication complex (pre-RC) is assembled. The pre-RC is assembled in G_1_ and includes the ORC, Cdc6, Ctd1, and the replicative DNA helicase, Mcm2–7. Early during S-phase, the pre-RC is phosphorylated by cyclin-dependent kinases. This event results in the formation of active replication fork(s) by the recruitment of Cdc45, Mcm10, and GINs complex, which constitute the CMG helicase (reviewed in [14]). Next, the DNA polymerase alpha (Polα) containing complex, Polα-primase, synthesizes short RNA-DNA primers on both the leading and lagging strand [15,16] to establish an actively synthesizing replication fork, Figure 1.

The movement of the replication fork is driven by the CMG helicase complex, which unwinds the DNA double helix. Single-stranded DNA binding protein, replication protein A (RPA) [17,18,19,20], coats and stabilizes single-stranded DNA (ssDNA) formed at the replication fork (structural and functional studies are reviewed in [21]). After a single priming event close to the origin, leading strand synthesis occurs in a continuous fashion by Polε. Discontinuous synthesis of the lagging strand is initiated at intervals of approximately 150 nucleotides by the Polα-primase complex which synthesizes short RNA-DNA primers [22]. These primers are subsequently extended by Polδ. The processivity of both Polδ and Polε are increased by proliferating cell nuclear antigen (PCNA), which encircles the DNA template and tethers replicative DNA polymerases to the template DNA (PCNA functions reviewed in [23]). Additional details about the structure and subunits of Polδ and Polε can be found in references [24,25,26,27,28,29,30]. Replication factor C (RFC) acts to load PCNA onto DNA at the replication fork [19,31]. Once Polδ finishes synthesis of each Okazaki fragment and begins strand displacement synthesis into the downstream RNA/DNA primer, flap endonuclease Rad27 (human FEN1) and nuclease/helicase Dna2 (human DNA2) act to remove flaps created by Polδ (the roles of nucleases during Okazaki fragment maturation are reviewed in [32]). The nicks created by flap removal are repaired by DNA ligase (reviewed in [33]) resulting in a continuous lagging strand. In addition to their primary roles at the replication fork described here, many of these proteins have additional functions in replication and repair, which are often regulated by post-translational modifications.

The assignment of polymerases to opposite strands was first supported by evidence that Polδ and Polε proofread errors on opposing strands [34]. Additionally, yeast strains lacking Polδ exonuclease function are not viable in combination with loss of Rad27 [35], and Polδ is capable of using its exonuclease function to maintain a ligatable nick during strand displacement reactions [36], which indicates Polδ has a role in processing Okazaki fragments on the lagging strand. Furthermore, biochemical studies have shown that the CMG helicase interacts with and stabilizes Polε, but not Polδ, on leading strand-like templates in vitro [37]. Recently, Polδ variants [38] and Polε variants [39] that produce biased error rates have been used in conjunction with whole-genome sequencing (WGS) to demonstrate Polε and Polδ synthesis results in errors on the leading and lagging strand, respectively [40]. In contrast to the commonly accepted model, a number of observations reviewed in [41] support a model in which Polδ takes over synthesis on the leading strand after Polε synthesis is impeded. Although the current consensus is that Polδ and Polε are equally responsible for synthesis of nearly the entire genome, some evidence indicates that approximately 1.5% of the mature genome results from Polα synthesis [42]. Several mutations affecting the catalytic subunit of Polα increase the mutation rate in yeast lacking MMR or Polδ proofreading, which further indicates that the mature genome contains DNA synthesized by Polα [43,44]. Although most knowledge pertaining to the roles of replicative polymerases at the replication fork is the result of studies utilizing yeast models and in vitro biochemical studies, recent next-generation sequencing of human tumors with Polε exonuclease domain mutations indicates that the organization of the human replication fork may be similar [45]. These studies have found that Polε-induced mutations occur asymmetrically with respect to direction of replication in a pattern consistent with Polε primarily synthesizing on the leading strand. Additional work using defined experimental systems are needed to determine if current models of the replication fork based on yeast studies accurately depict the architecture of the human replication fork and strand-specific roles of DNA polymerases.

Error-prone translesion synthesis (TLS) polymerases can also synthesize DNA during DNA replication, although their roles are limited to rare circumstances. In yeast models, DNA polymerase zeta (Polζ) can carry out synthesis at the replication fork to bypass lesions that stall Polδ and Polε (reviewed in [46]) and participates in DNA replication under circumstances of replication stress or defective replication [47,48]. In human cells, TLS polymerase eta (Polη) participates in immunoglobulin hypermutation [49], and recent evidence indicates that Polη may contribute to synthesis of regions of the genome that are difficult to replicate [50]. The contribution of TLS enzymes to DNA synthesis at the replication fork in the absence of exogenous DNA damage has not been studied in detail in human cells. Based on the error-prone nature of these polymerases, they may contribute to replication-associated mutagenesis in difficult to replicate genomic regions and under conditions known to commonly cause replication stress in tumor cells.

Upon encountering obstacles to replication (e.g., DNA lesions, DNA secondary structures, and elongating transcription complexes), additional protein factors are recruited to stalled forks to help maintain their integrity. Such factors include the RecQ helicases, BLM (Bloom’s Syndrome helicase), WRN (Werner’s Syndrome helicase), RECQ5 (RecQ-like protein 5), and RECQ1 (RecQ-like protein 1) and DNA translocases, SMARCAL1 (SWI/SNF Related, Matrix Associated, Actin Dependent Regulator of Chromatin, Subfamily A Like 1), ZRANB3 (Zinc Finger RANBP2-Type Containing 3), and HLTF (helicase-like transcription factor) that are thought to limit undesirable recombination at stalled forks and facilitate replication restart (reviewed in [51,52,53,54,55,56,57,58]). Deficiency in these factors results in increases in genome instability as indicated by persistent DNA breakage, RAD51 foci, and in many cases sister chromatid exchanges [59,60,61]. Individuals inflicted with Werner’s Syndrome (deficiency in WRN helicase), Bloom’s Syndrome (deficiency in BLM helicase), Schimke immuno-osseous dysplasia (deficiency in SMARCAL1), or germline mutations in the *RECQL* gene display elevated incidence of cancer [62,63,64,65], suggesting that the genome instability associated with these defects can lead to cancer-promoting genetic alterations. In contrast, these proteins also appear to support continued replication in rapidly proliferating cancer cells. RecQ helicases are often over-expressed within sporadic human tumors where they likely relieve some oncogene-induced replication stress (reviewed in [66]). Accordingly, depletion of these factors or SMARCAL1 sensitizes cancer cells to chemotherapeutics and can inhibit cancer cell growth [63,67], indicating that targeting these factors may be a powerful cancer therapy.

### 1.2. Mismatch Repair Deficiencies Promote Cancer

Before the genetic nature of cancer was fully appreciated, Lawrence Loeb authored an article entitled “Errors in DNA Replication as a Basis for Malignant Change” in which the authors predicted that cancer might result from altered DNA polymerases that cause more errors during DNA replication and repair [68]. Numerous observations since then have supported this theory. Most significantly, decades of research examining mismatch repair defects have made it clear that errors originating from DNA synthesis contribute to carcinogenesis.

MMR is a highly-conserved pathway that acts to fix errors made during DNA replication. Eukaryotic MMR begins when a mismatch or insertion/deletion mispair is recognized by MutSα or MutSβ. MutSα is composed of Msh2 and Msh6 and recognizes base-base and small (one or two base) insertion/deletion mispairs. MutSβ is composed of Msh2 and Msh3 and recognizes small and large insertion/deletion mispairs, but not base-base mispairs. Once MutSα or MutSβ is bound to a mismatch, it recruits MutLα, composed of Mlh1 and Pms1 in *S. cerevisiae* and MLH1 and PMS2 in humans. MutLα acts as an endonuclease, which nicks the strand to be excised and directs the activities of other proteins in subsequent steps. The DNA strand containing the mismatch is excised by the action of Exo1 and the resulting gap is filled by the actions of RPA, RFC, PCNA, and Polδ [69,70]. In yeast, deletion of genes encoding MMR proteins increase forward (*CAN1*) mutation rates 18- to 40-fold and the rate of frameshifts in homopolymeric runs measured by reversion reporters as much as 662-fold [71]. Elevated spontaneous mutagenesis caused by MMR defects has been observed in many model systems (reviewed in [72]). Defects in MMR also drastically increase the frequency of cancer in mice, reviewed in [73]. In humans, inherited mutations in MMR genes predispose to colorectal cancer (CRC) in Lynch syndrome [74,75]. Additionally, MMR genes are inactivated via hypermethylation in approximately 15% of sporadic CRC, endometrial (EC), and gastric cancers (reviewed in [76]). The use of next-generation sequencing has shown that tumors with MMR defects are commonly hypermutated. For example, in colorectal cancer a distinct set of hypermutated tumors have on average 12-fold more non-silent mutations within sequenced exomes, compared to non-hypermutated CRC tumors. The majority of these hypermutated tumors had either silencing of *MLH1* or somatic mutations in MMR genes and displayed microsatellite instability [77]. Tumors with MMR deficiencies have high numbers of short (<3 base pair) insertions and deletions at mono- and polynucleotide repeats and cancer-associated mutational signatures 6, 15, 20 and 26 [78,79]. Common to these MMR mutation signatures are a high probability of C-to-T, C-to-A, and/or T-to-C base substitutions. Each MMR defect-associated mutation signature has multiple preferred trinucleotide sequences in which specific mutations tend to occur [78,79].

### 1.3. Mutagenic Human Replicative Polymerase Variants Give Rise to Cancer

The base selectivity and proofreading activities of replicative DNA polymerases act in series with MMR to avoid replication errors and reduce the likelihood of mutation [80]. The combination of MMR defects and mutations that lower replicative polymerase fidelity cause a synergistic increase in mutagenesis that often results in lethality due to a rapid accumulation of mutations [3,4,80,81,82]. Recently, multiple studies have provided three lines of evidence that indicate defects in replicative polymerases promote carcinogenesis by increasing mutation rates: (1) mutations in genes encoding the enzymatic subunits of human Polδ and Polε, *POLD1* and *POLE* respectively, predispose to hereditary CRC; (2) a significant number of Polε variants have been found in sporadic, MMR-proficient, hypermutated human tumors; and (3) studies of Polδ and Polε variants found in both hereditary and sporadic CRC using genetic model systems and biochemical approaches indicate that these polymerase variants elevate the spontaneous mutation rate.

Efforts to find novel causes of hereditary CRC using next-generation sequencing found that rare germline *POLD1* and *POLE* mutations predispose individuals to CRC [83]. This study found a perfect linkage between the *POLD1*-*S478N* and *POLE-L424V* mutations and CRC among multiple members of affected families and identified *POLD1-P327L* as an additional variant likely to be pathogenic [83]. In addition, 39 tumors from individuals with the germline *POLE* mutation, *POLE-L424V*, were screened for mutations in six proto-oncogenes and tumor suppressors. All the driver mutations found were base substitutions, many of which were concentrated at atypical hotspots [83]. Because error-prone replicative polymerase variants produce mutational spectra dominated by base substitutions, the previous observation indicates that the Polδ and Polε variants encoded by the germline *POLD1* and *POLE* alleles generate driver mutations in these patients. Since this seminal discovery, several publications have found evidence supporting roles for additional germline *POLD1* and *POLE* mutations in cancer predisposition, in which carriers typically develop multiple adenomas, polyposis, CRC, and/or EC. These pathogenic germline mutations in *POLE* and *POLD1* mutations are summarized in Table 1. Several recent publications indicate that some inherited Polε variants may give rise to significantly different diseases. A 14-year-old boy with polyposis and rectosigmoid carcinoma was found to have inherited a *POLE-V411L* mutation [84]. Because this case clinically resembled inherited bi-allelic mismatch repair deficiency in its early onset and severity, it appears that different polymerase variants may have more severe phenotypes. Unlike the aforementioned *POLD1* and *POLE* mutations, *POLE-W347C* may predispose to cutaneous melanoma and affected patients do not have CRC or EC [85].

Cancer genome sequencing projects have also identified somatic changes in the exonuclease domain of Polε in approximately 3% of sporadic CRC tumors and 7% of sporadic EC tumors [77,96,97,98]. Because these *POLE* exonulease domain mutations are found primarily in tumors that do not have microsatellite instability and are hypermutated, the current consensus is that the encoded Polε variants are responsible for the high number of mutations found in these tumors and are pathogenic, Table 1, and reviewed in [99,100]. Tumors with known pathogenic *POLE* mutations represent a separate class of tumors due to the number of mutations present. The density of mutations in hypermutated CRC cancers with MMR deficiencies is approximately 12 to 55 mutations per 10^6^ base pairs. In contrast, hypermutated tumors with *POLE* variants have mutation densities ranging from approximately 60 to 380 mutations per 10^6^ base pairs, and are thus termed “ultra-mutated” [77]. Because next-generation sequencing methods employed in these studies only detect near clonal mutations and not mutations present in individual tumor cells, these mutation densities likely grossly under-estimate the total number of mutations caused by *POLE* variants within tumors.

Several lines of evidence indicate that germline and somatic *POLE* and *PODL1* variants increase cancer predisposition by elevating mutation rates. For *POLD1* variants that predispose to CRC, mutations affecting residues homologous to D316 and L474 [87] and S478 [83] were previously shown to increase mutagenesis in yeast models. The most common *POLE* mutation found in sporadic CRC and EC, P286R, was found to increase the mutation rate when modeled yeast [89]. Inexplicably, the increase in the mutation rate caused by the analogous mutation in diploid yeast was approximately 300-fold greater than that caused by a mutation eliminating Polε proofreading [86,91,92,94]. In contrast, four human single nucleotide polymorphisms (SNPs) modeled in yeast, *pol3-K855H*, *pol3-K1084Q*, *pol2-F709I*, and *pol2-E1582A* did not change the rate of spontaneous mutagenesis [101]. In addition, the cancer-associated human Polε variants (P286R, P286H, F367F, S459F, and L424V) have been shown to have reduced exonuclease activity and higher error rates in vitro using LacZ gap-filling assays [45]. Together these studies indicate that a subset of replicative polymerase variants found in human cancers promote carcinogenesis by increasing mutation rates in vivo.

Much work remains to be done before a comprehensive understanding of the role that replicative polymerase variants play in promoting cancer can be realized. Recent efforts to sequence cancer genomes have led to the discovery of least 346 unique mutations in *POLE* alone (cataloged within the cBioPortal data sets, http://www.cbioportal.org, [102,103]). Additionally, the number of *POLD1* and *POLE* mutations in human cancers will likely increase substantially as more cancer genomes are sequenced. A major challenge going forward will be to differentiate the few polymerase variants that reduce replication fidelity and promote cancer from the large number of randomly occurring passenger mutations within *POLE* and *POLD1*. Next-generation sequencing of sporadic endometrial and colorectal tumors have made it clear that *POLE* exonuclease domain mutations (EDMs) are causative in a subset of hypermutated, microsatellite stable (MSS) tumors (reviewed in [99,100]). Based on these findings, it would seem prudent to study those somatic *POLE* mutations that fall within the exonuclease domain and are found in MSS hypermutated tumors. However, compelling results from [101,104] suggest that less frequent somatically occurring, cancer-associated *POLD1* mutations outside of the exonuclease domain found in MMR deficient tumors have the potential to elevate mutation rates and promote cancer. Therefore, solely focusing upon *POLE* and/or EDMs may fail to identify all the replicative polymerase variants that contribute to cancer etiology. Consequently, most current efforts to identify pathogenic germline *POLD1* and *POLE* mutations have focused solely on the exonuclease domain [86,90,91,92,93,94]. The most direct and definitive method to assess the pathogenicity of cancer-associated polymerase variants is to determine if they elevate mutation rates in human cell lines. Surprisingly, no studies have been published that show any cancer-associated polymerase variant increases mutation rates in cultured human cells.

Several interesting conundrums exist in respect to mutagenic polymerase variants and cancer. First, it is unclear why hypermutated tumors with *POLE* exonuclease domain mutations have better survival than other tumors of the same cancer type. Although it is easy to imagine that hypermutated tumors would be more resistant to chemotherapies due to increased tumor heterogeneity, in fact the opposite appears to be true. Results from a recent study indicate that tumors hypermutated by mutant Polε may invoke a stronger immune response [105], which may explain this contradiction. Alternatively, the extremely high mutation load within these tumors may place a fitness burden on these tumors. Second, it is unclear why error-prone replicative polymerase variants tend to give rise to a limited number of tumor types. Third, it is unknown why almost all sporadic polymerase variants that give rise to hypermutated tumors are within the exonuclease domain of Polε. Mutations that decrease or eliminate exonuclease function might be more prevalent than specific mutations that decrease base selectivity. Finally, given that the exonuclease domains of Polδ and Polε are a similar size, share a great deal of homology, and that both polymerases synthesize similar amounts of DNA during replication, why *POLE* mutations are almost exclusively found as promoters of sporadic cancer is unclear. It has been speculated by others that proofreading-deficient Polδ variants might lead to a more severe phenotype due to their propensity to elevate frameshift mutations in addition to base substitutions and are therefore selected against in cells. However, because germline *POLD1* mutations that likely decrease, or eliminate exonuclease function give rise to hereditary CRC, it is unlikely that similar somatic mutations would be selected against. One possibility is that during sporadic tumorigenesis, human cancer cells require Polδ exonuclease for functions needed to cope with DNA damage resulting from replication stress and elevated levels of reactive oxygen species and Polε exonuclease function is dispensable for these functions.

### 1.4. Damage to Single-Stranded DNA on the Lagging Strand Template Causes Mutation in Cancer

In addition to deficiencies in mismatch repair and polymerase exonuclease activity generating mutations during replication, recent evidence has highlighted increased damage in ssDNA formed on the lagging strand template as an important source of replication-associated mutagenesis. In human cancers, this is exemplified by the mutagenic activity of APOBEC cytidine deaminases. Eleven AID/APOBEC family members are encoded in the human genome, of which, seven are APOBEC3 members [106] (Table 2). APOBECs are involved in several normal biological processes including roles in lipid metabolism and immune function (e.g., antibody maturation and inhibiting viral propagation) [106]. The APOBEC3 enzymes (A3) mediate their cellular effects by catalyzing the sequence-specific deamination of deoxycytidines to deoxyuridines within single-stranded nucleic acids [107,108,109,110]. Most APOBECs target the trinucleotide sequences TCA and TCT (hereafter referred to jointly as TCW) [106]. Their C-to-U editing functionality can ultimately result in either C-to-T transitions or C-to-G transversions depending on the efficiency by which uracil glycosylase activity converts deamination-induced deoxyuridines to abasic sites and the choice of DNA polymerase inserting nucleotides across from the abasic sites [111].

While APOBECs are typically tightly regulated by controlled expression [119] and cellular localization to the cytoplasm [120], deleterious consequences can result when off-target editing of the host’s genome occurs. Accordingly, emerging data indicate that APOBECs play a role in the etiology of many human cancers. An overabundance of APOBEC signature mutations (C-to-T and C-to-G substitutions in TCW sequences) have been found in ~15% of sequenced tumor samples [78]. APOBEC-mutagenized tumors frequently display mutation densities up to 50 mutations per 10^6^ bp [121,122], indicating that like MMR and replicative polymerase defects, APOBEC-derived mutagenesis is a process that litters the genomic landscape with somatic point mutations. Cumulative evidence has shown that APOBEC expression causes a mutator phenotype with a positive correlation between increased APOBEC mRNA expression and the extent of APOBEC mutagenesis [121,122,123]. The nucleotide context of mutational signatures, their genomic distribution, and regions of localized hypermutation (termed kataegis) found in studies characterizing APOBEC activity in model systems are extremely similar to those observed in human tumors, suggesting that APOBEC activity potentially contributes to the onset and/or progression of tumor formation by increasing the mutational burden (reviewed in [124]). Additionally, bioinformatics analyses by Henderson et al. revealed that the proto-oncogene, *PIK3CA*, was frequently mutated in tumor types expressing high APOBEC mRNA levels such as HPV-positive CESC and HNSCC (cervical squamous cell carcinoma and endocervical adenocarcinoma and head and neck squamous cell carcinoma) [125]. Moreover, 88% of these *PIK3CA* mutations occurred in two hotspot sites occurring at APOBEC-targeted sequences (TCW) in the helical domain of the protein that binds the p85 inhibitory protein, as opposed to the more common activating kinase domain mutation which does not occur at an APOBEC target sequence. This evidence strongly indicates that in some capacity, APOBEC enzymes contribute to the mutations selected for during cancer development. In accord with these observations, over-expression of APOBEC1 and APOBEC2 in mice has been shown to be sufficient to induce tumorigenesis, suggesting that unrestrained activity of this family of enzymes is carcinogenic [126,127]. However, no elevation of mutation was detected in APOBEC2-induced tumors and mutagenesis in mouse tumors induced by APOBEC1 were not evaluated leaving the mechanism of this tumorigenesis unclear.

Determining the identity of the APOBECs that mediate cancer mutagenesis has been a recent focus of the field. APOBEC3A (A3A) and APOBEC3B (A3B) have nuclear localization capabilities, making them likely candidates for genomic DNA editing [120,128]. Experimentally, A3B was shown to be over-expressed, the primary source of cytidine deaminase activity, and a source of mutation in a panel of breast carcinoma cell lines, indicating a role for this enzyme in breast cancer mutagenesis [129]. Similarly, additional bioinformatics analyses found that A3B mRNA expression levels positively correlate with the amount of APOBEC signature mutations in multiple tumor types including breast, bladder, cervix, head and neck, and lung (adenocarcinoma and squamous cell carcinoma) [121,122]. Recently, a human polymorphism upstream of the *APOBEC3A* gene and linked to bladder cancer risk, was shown to increase A3B expression, suggesting that greater amounts of this enzyme in cells may be carcinogenic [130]. However, seemingly paradoxical, a germline APOBEC3A-APOBEC3B fusion polymorphism causing deletion of A3B is associated with greater risk for breast, ovarian and liver cancer along with an overall increase in mutations present in *ΔA3B^−/−^* breast cancers [130,131,132,133,134,135]. One potential explanation for this is that the deletion of A3B results in increased activity of other APOBEC enzymes, perhaps in a compensatory fashion. Caval et al. [131] studied the consequences of the fusion of the A3B-3′UTR to A3A, which occurs in individuals containing the A3B deletion polymorphism. They found that the replacement of the A3A-3′UTR with that of A3B’s resulted in stabilization of A3A mRNA, increased A3A expression, and genomic DNA editing by A3A [131]. Supporting a role for A3A in cancer mutagenesis, Chan et al. [136] determined that when expressed in yeast, A3A and A3B preferred slightly different DNA sequences, targeting YTCA and RTCA, respectively. They further showed that A3A-like (YTCA) mutations were more abundant than A3B-like (RTCA) mutations in many sequenced tumors [136]. In addition to A3A and A3B activity, other APOBECs have been linked to cancer development. AID’s role in promoting cancers of the blood has been long established (reviewed in [137]), while APOBEC1 over-expression has been linked to the onset of esophageal adenocarcinomas [78,138]. Recent work by Reuben Harris and colleagues now suggests that A3H-I haplotype activity may account for some of the APOBEC-induced mutation load based on A3B-null breast tumor analysis [139].

Since APOBEC enzymes are ssDNA specific, determining the source of their substrate in a double-stranded genome has been a matter of great interest. Several candidate metabolic processes expose ssDNA for APOBEC mutagenesis, including transcription, DNA repair and DNA replication. Transcription-associated ssDNA was originally believed to be the main target of APOBEC activity, primarily by extension of AID’s known dependence on transcription to mediate somatic hypermutation and class switch recombination during B-cell maturation [112,113,114,115]. In fact, the expression of lamprey APOBEC, as well as hypermutator forms of AID and APOBEC3G (A3G) in yeast revealed an overabundance of mutations occurring mostly 5′ of transcription start sites, indicating that transcription intermediates can be targets of these enzymes [140,141]. Such damage to transcription bubbles could be very significant to human cancer mutagenesis as oncogene activation can lead to the elevated formation of R-loops as transcription becomes upregulated [142].

Similarly, the formation of kataegic events linked to the ectopic expression of AID, A3A, A3B, and A3G in yeast is dependent on Ung1 activity, indicating that DNA repair intermediates can provide substrates for these enzymes as well [116]. DNA double-strand break (DSB) repair intermediates may provide the greatest amount of substrate for kataegis, as these events are greatly elevated by induction of DSBs. Homology-directed repair of DSBs provides large stretches of ssDNA through 5′ to 3′ double-strand break resection [143,144], which APOBECs likely can mutagenize. Additionally, break-induced replication (BIR), a variant of homologous recombination involving only one end of a DSB, creates a very long ssDNA intermediate during the extended D-loop synthesis used to repair these breaks [145]. This synthesis is a form of conservative replication that has been shown to serve as a source of kataegis induced by alkylating DNA damage and presumably APOBEC enzymes as well [146].

Despite these links describing APOBEC mutagenesis of transcription and DSB repair processes, results from several studies indicate that most APOBEC-induced mutations occur during DNA replication in cancer genomes. Single-stranded DNA formed on the lagging strand template during Okazaki fragment synthesis provides the most abundant source of ssDNA during normal cell division, Figure 1. Moreover, establishment of bi-directional replication forks results in ssDNA in the lagging strand template occurring on different DNA strands dependent on the direction of fork movement. Multiple analyses of the distribution of APOBEC-induced mutations identified by WGS have utilized the asymmetry in the location of lagging strand-associated ssDNA to correlate the substitution patterns of APOBEC mutagenesis with replication-associated ssDNA. Bhagwat et al. [147] expressed the catalytic domain of human A3G in *E. coli* defective for repair of uracil (*ung* mutant) and determined that C-to-T substitutions induced by this enzyme preferentially occurred in replichore 1, while G-to-A substitutions occurred more frequently in replichore 2 of the genome. As replichore 1 and replichore 2 are replicated in clockwise and anticlockwise directions respectively, this distribution is consistent with cytidine deamination occurring predominantly in ssDNA on the lagging strand template [147]. No mutational strand bias was observed in relationship to transcriptional direction, indicating that in replicating cells, the primary substrate for A3G mutagenesis is ssDNA at the replication fork. The authors saw a similar phenomenon with spontaneous mutagenesis, indicating that mutagenesis associated with damage to ssDNA at the fork may be a general source of mutation beyond APOBEC activity. In concert with this finding, other APOBECs likewise have been experimentally shown to prefer replication-based substrates. In yeast ectopically expressing A3A or A3B, strand-biased mutations were observed in gene mutation reporters placed on either side of a single autonomously replicating sequence (ARS). Through WGS, the pattern of mutagenesis identified was indicative of replicative asymmetry across the genome as there was a predominance of G-to-A substitutions 5′ of origins and C-to-T substitutions 3′ of origins [117]. As with A3G mutation in *E. coli*, neither A3A- nor A3B-induced mutations in yeast displayed significant transcriptional strand asymmetries, indicating that both of these APOBECs predominately mutate replication intermediates and that this preference is generally applicable to the entire APOBEC family. Supporting this, even forced S-phase expression of AID, an APOBEC whose mutagenic capacity is undeniably linked in transcription, results in increased cell death, suggesting that this enzyme may also be able to deaminate replication-associated ssDNA if it is available [148]. APOBEC deamination of replication intermediates has also been reported in human cells where it is a source of DSBs produced by S-phase expression of A3A [118]. These experimental analyses have since served as crucial support that during tumor development, APOBECs likely mutagenize cancer genomes by taking advantage of the highly proliferative nature of these cells. WGS of hundreds of samples across multiple tumor types indicate that, as in yeast and *E. coli* expressing APOBEC enzymes, APOBECs predominantly deaminate the lagging strand template in human tumors. While the locations of origin of replication in human cells are largely unknown, the direction of replication across individual regions of the genome can be inferred from replication timing profiles. Using this information, three groups have profiled the position of APOBEC signature mutations in relationship to replication directions, uncovering a significant elevation of C substitutions in regions replicated with rightward moving forks, while G substitutions occurred predominantly in regions replicated with leftward moving forks. This “replicative asymmetry” (also termed “R-class”) is consistent with mutagenesis of the ssDNA lagging strand template and has been observed for other mutation signatures associated with replication defects (i.e., MMR defects and Polε mutations). Only limited transcriptional asymmetry was observed among APOBEC-induced mutations, in contrast to UV and tobacco smoke-induced mutations whose localization is lessened on the transcribed strand of genes by transcription coupled repair [149,150,151].

Despite the significant advances in understanding the roles of APOBEC enzymes in tumor mutagenesis, multiple questions remain. While it is generally accepted that these enzymes are responsible for the production of large numbers of mutations in cancer, in many cases the initiating events leading to their dysregulation are still unknown. The association of APOBEC mutagenesis with cervical and head and neck cancers [78,121,122,125], which frequently involve HPV infection, suggest that up-regulation of these enzymes by HPV or induction of replication stress by HPV encoded proteins that inhibit RB1 function [152,153] may be a key event in initially establishing an APOBEC mutator phenotype. However, the cellular events that cause mutagenesis resulting from aberrant APOBEC activity in other tumor types is unknown. Understanding the root causes of APOBEC dysregulation is likely to provide key insights into the tumor specificity of these enzymes. Similarly, the biological effects of APOBEC mutagenesis on cancer development, progression, and treatment are largely unclear. The association of APOBEC polymorphisms with cancer risk and the apparent APOBEC-induction of *PIK3CA* mutations [125] indicate that these enzymes likely play significant roles in promoting cancer onset. However, the large numbers of mutations these enzymes induce suggests that they may additionally contribute to continued evolution of the tumor and ultimately to therapy resistance. This role is supported by experimental evidence indicating that elevated A3B expression in breast cancer cell lines increases the resistance of derived xenografted tumors to the drug tamoxifen [154]. Intriguingly, APOBEC mutagenesis within tumors may not solely provide deleterious effects. Similar to tumors with polymerase defects that also produce high mutation loads, high numbers of APOBEC mutations in bladder cancer associate with longer patient survival times [130]. This suggests that the activity of these enzymes may reduce the overall fitness of cancer cells. This effect may enable the development of future therapeutic strategies that take advantage of liabilities associated with APOBEC activity.

### 1.5. Future Directions: Tumor Specific Metabolic Changes as Modulators of Replication Fork-Associated Mutagenesis

We speculate that the rate of mutagenesis resulting from activities at the replication fork may be affected by mutations that activate oncogenes and inactivate tumor suppressors. Consequently, mutation rates in tumor cells might fluctuate throughout the process of carcinogenesis. In addition, mutations present in tumor subpopulations as both drivers and passengers that increase the rate of mutation could allow tumors to acquire mutations needed for progression and resistance to therapies while allowing most cells to escape the deleterious effects of an ultra-high mutation rate.

Pathways that regulate dNTP levels are often mutated in human cancers. Mutations that activate the Ras signaling pathway decrease dNTP pools by decreasing levels of RRM2, a subunit of human ribonucleotide reductase (RNR) [155]. Loss of the retinoblastoma tumor suppressor (RB) causes elevated expression of many genes involved in dNTP metabolism and an elevation of dNTP pools [156]. AMP-activated protein kinase (AMPK) activity is often deregulated in cancer. AMPK regulates phosphotransferase nucleoside diphosphate kinase (NDPK), which is the enzyme responsible for converting dNDPs to dNTPs [157]. The proto-oncogene *MYC* (C-Myc) is overexpressed in most human tumors. Overexpression of C-Myc in normal human cells leads to increased expression of thymidylate synthase (TS), inosine monophosphate dehydrogenase 2 (IMPDH2) and phosphoribosyl pyrophosphate synthetase 2 (PRPS2) and increased dNTP pools [158]. Tumor suppressor p53 restricts human RNR activity by binding to human RNR regulatory subunits RRM2 and p53R2 [159]. Taken together, the results of these studies indicate that dNTP pools likely fluctuate during the process of carcinogenesis.

In respect to polymerases acting at the replication fork, both decreased and increased dNTP levels have been shown to decrease replication fidelity. In yeast, decreasing dNTP pools by exposure to the ribonucleotide reductase inhibitor, hydroxyurea, results in an increase in mutagenesis that is primarily Polζ dependent [48]. Conversely, in vitro experiments have shown that increasing dNTP concentration both improves the likelihood that a replicative polymerase will extend from a mismatched primer terminus [160,161,162], and increases errors during synthesis [163]. Consistent with these findings, proportional increases in dNTP levels in *E. coli* are also mutagenic [164,165]. Furthermore, several studies in yeast have shown that moderately decreasing dNTP levels in yeast by deletion of *DUN1*, suppresses the mutator phenotype of both Polδ and Polε variants [4,81,163,166]. Taken together, these results from biochemical experiments and model systems indicate that changes in dNTP levels which occur during carcinogenesis likely substantially modulate mutagenesis caused by polymerase variants in human tumors.

We speculate that several phenomena occurring during carcinogenesis may modulate APOBEC-induced mutagenesis at the replication fork. First, oncogene activation and elevated DNA damage during cancer development can cause replication stress that increases the formation of replication-associated ssDNA [167]. Such increases in ssDNA availability may provide greater opportunities for APOBECs to damage the chromosomes of proliferating tumor cells, resulting in dramatically higher APOBEC-induced mutation densities. Recent evidence suggests that synergistic interactions between APOBEC mutagenesis and replication stress may occur through two mechanisms. First, replication stress appears to increase the expression level of A3B in a variety of cancer cell lines, thereby increasing the cellular pool of this mutator [168]. Secondly, a greater mutagenic response was observed for both A3A and A3B expressed in yeast in the presence of the replication inhibitor hydroxyurea (HU) as well as in strains lacking replication fork stability proteins [117]. Mutation spectra indicate that the observed increase in mutagenesis likely occurred due to more replication-associated ssDNA being available on both the leading and lagging strand during DNA replication. Consequently, cancer-associated mutations that result in replication stress by decreasing ribonucleotide reductase expression [155] could increase APOBEC-induced mutagenesis. Although speculative, in cells in which oncogene activation leads to increased replication stress caused by elevated replication origin firing, dNTPs levels could also become insufficient for efficient replication and result in more ssDNA being available to APOBECs. Genetic and epigenetic differences that influence dNTP levels in tumor cells should be studied as a possible explanation for why tumors with similar APOBEC expression levels have drastically different amounts of APOBEC-signature mutations. The extent to which the synergistic interactions between replication stress and APOBEC activity impact the abundance of mutations in tumors remains unclear.

## 2. Conclusions

In humans, each cell division requires the replication of approximately 3.3 × 10^9^ base pairs of DNA. Current estimates indicate the number of cells in the human body is around 3.72 × 10^13^ [169], and approximately 5 × 10^10^ to 7 × 10^10^ cells are replaced daily. Taken together the amount of DNA that is replicated over a human lifetime is staggering. Fortunately, genomic stability is typically maintained by a semi-conservative process for DNA replication with multiple mechanisms that increase fidelity such that typically less than one mutation occurs per cell division [2]. Although multiple processes safeguard the fidelity of DNA replication, there are still inherent risks involved in this necessary process. Mutations generated during DNA replication promote carcinogenesis by inactivating tumor suppressors and activating oncogenes. Recent developments have made it apparent that the risks associated with DNA replication are increased by specific mutations in replicative polymerases that promote carcinogenesis. Furthermore, ssDNA produced by the process of DNA replication represents a potential risk for mutagenesis mediated by chemicals and enzymes. Consequently, targeting of replication-associated ssDNA by APOBEC enzymes, whose activity is dysregulated in some cancer cells, results in significant mutagenesis in many tumors. Replication-associated mutagenesis both promotes carcinogenesis and likely affects clinical outcomes by increasing tumor heterogeneity. Further characterizing mutations and pathways that modulate risks associated with DNA replication will provide a better understanding of the etiology of cancer-causing mutations and may provide future opportunities for cancer treatment.

## Figures and Tables

**Table 1:** Pathogenic replicative polymerase mutations.

Amino Acid Change ^1^	Somatic/Germline	Cancer Type ^2^ (n) ^3^	Mutator Phenotype in Yeast [References]	Biochemical Support/Enzyme [References]
*POLD1-*				
*D316G*	Germline [86]	CRC, EC, and breast	Yes [87]	Yes/T4 polymerase [88]
*D316H*	Germline [86]	CRC and breast	Yes [87]	Yes/T4 polymerase [88]
*P327L*	Germline [83]	None, patient had multiple colonic adenomas	Yes ^5^ [89]	Yes/human Polε [45]
*R409W*	Germline [86]	CRC	N.d.	N.d.
*L474P*	Germline [86]	CRC and EC	Yes [87]	Yes/human Polε [45]
*S478N*	Germline [83]	CRC and EC	Yes [83]	N.d.
*POLE-*				
*W347C*	Germline [85]	Cutaneous melanoma	Yes [85]	N.d.
*N363K*	Germline [90]	CRC and EC	N.d.	N.d.
*D368V*	Germline [91]	CRC	N.d.	Yes/T4 polymerase [88]
*P436S*	Germline [92]	CRC	N.d.	N.d.
*Y458F*	Germline [93]	CRC	N.d.	Yes/T4 polymerase [88]
*L424V/I*	Both [83]	Hereditary CRC, EC (2) ^4^, breast (1) ^4^	Yes ^6^ [87]	Yes/human Polε [45]
*P286R/L/H*	Somatic	CRC (5), EC (10), breast (1), stomach (1), pancreas (1)	Yes [89]	Yes/human Polε [45]
*F367S*	Somatic	CRC (1)	N.d.	Yes/human Polε [45]
*V411L*	Both [84]	CRC (3), EC (6), stomach (1)	N.d.	Yes/human Polε [45]
*S459F*	Somatic	CRC (4)	N.d.	Yes/human Polε [45]
*S297F*	Somatic	EC (1), cervical (1)	N.d.	N.d.
*P436R*	Somatic	CRC (1)	N.d.	Yes/human Polε [45]
*M444K*	Somatic	EC (1)	N.d.	N.d.
*A456P*	Somatic	EC (1)	N.d.	N.d.

Colorectal cancer (CRC), endometrial cancer (EC), not determined (N.d.). ^1^ The somatic *POLE* exonuclease domain mutations listed have been implicated in CRC and EC tumorigenesis due to their presence in hypermutated MSI-stable and MSI-low tumors. The *POLE* and *POLD1* mutations that predispose to CRC are from references [83,84,86,90,91,92,93,94]; ^2^ The incidence of mutations in different types of sporadic tumor (n) is from cBioportal and summarizes TCGA provisional data and those from published studies from other institutes; ^3^ For a more detailed account of incidence of germline *POLE* and *POLD1* mutations and patient phenotype, please see [95]; ^4^ Though *POLE-L424V* is the most common mutation that predisposes to CRC, one EC and one breast cancer tumor with the L424V mutation are not hypermutated; ^5^ Evidence for these alleles producing a mutator phenotype is inferred from studies of yeast Polε; ^6^ Evidence for these alleles producing a mutator phenotype is inferred from studies of yeast Polδ.

**Table 2:** APOBEC characteristics and their involvement in cancer mutagenesis.

APOBEC Family Member	Mutation Motif Preference	Cellular Localization	Expression Correlates with TCW Mutations in Tumors	Evidence for Mutation during Transcription	Evidence for Mutation during Replication	Evidence for Mutation during DSB Repair	References
AID	WRC	Cytoplasmic	N/A	Yes	Yes	Yes	[112,113,114,115,116]
APOBEC1	TCW	Pan Cellular	N.d.	N.d.	N.d.	N.d.	˗
APOBEC2	N.d.	N.d.	N.d.	N.d.	N.d.	N.d.	˗
APOBEC3A	TCW	Pan Cellular	Yes	Limited	Yes	Yes	[116,117,118]
APOBEC3B	TCW	Nuclear	Yes	Limited	Yes	Yes	[116,117]
APOBEC3C	TCW	Pan Cellular	No	N.d.	N.d.	N.d.	˗
APOBEC3D/E	TCW	Cytoplasmic	No	N.d.	N.d.	N.d.	˗
APOBEC3F	TCW	Cytoplasmic	No	N.d.	N.d.	N.d.	˗
APOBEC3G	CC	Cytoplasmic	N/A	Limited	Yes	N.d.	[116]
APOBEC3H	TCW	Cytoplasmic	No	N.d.	N.d.	N.d.	˗
APOBEC4	N.d.	N.d.	N.d.	N.d.	N.d.	N.d.	˗

N.d. = Not determined; DSB = DNA Double Strand Break; W = A or T; R = A or G; Mutated base is underlined.
